# Case report: Interstitial pneumonitis after initiation of lamotrigine

**DOI:** 10.3389/fpsyt.2023.1203497

**Published:** 2023-07-03

**Authors:** Victoria Watzal, Godber Mathis Godbersen, Ana Weidenauer, Matthäus Willeit, Valentin Popper, Michael Treiber, Maximilian Preiss, Dominik Ivkic, Ulrich Rabl, Gernot Fugger, Richard Frey, Christoph Kraus, Dan Rujescu, Lucie Bartova

**Affiliations:** ^1^Clinical Division of General Psychiatry, Department of Psychiatry and Psychotherapy, Medical University of Vienna, Vienna, Austria; ^2^Comprehensive Center for Clinical Neurosciences and Mental Health, Medical University of Vienna, Vienna, Austria

**Keywords:** lamotrigine (LTG), interstitial pneumonitis, adverse events, case report, pulmonary condition

## Abstract

The second-generation anticonvulsant lamotrigine is widely used in the psychiatric field as a mood stabilizer or antidepressant augmentation therapy. Although particularly older anticonvulsants are known for their potential to cause hypersensitivity syndromes, newer antiepileptic drugs do hold a certain risk as well. Presenting a case of a 32-year-old male inpatient of African ethnicity suffering from a primary severe depressive episode in the course of a recurrent major depressive disorder, we report the occurrence of a rapid-onset drug-induced pneumonitis. Herewith, the interstitial pneumonitis occurred after the initiation of 25 mg lamotrigine as an augmentation therapy. Except for the clear temporal correlation between the administration of lamotrigine and the onset of pneumonitis, we did not reveal any further potentially causal diagnostic hints. Importantly, no relevant genetic variations of metabolizing enzymes or drug interactions resulting in lamotrigine overdosage as a potential cause of toxicity were identified. Our experience with a potentially life-threatening adverse drug reaction shortly after the initiation of the largely well-tolerated lamotrigine suggests a potential side effect under the second-generation anticonvulsant although similar adverse events are deemed to be very rare.

## 1. Introduction

Lamotrigine is an antiepileptic drug, primarily applied in the treatment of epileptic syndromes as a mono-therapeutic regimen or as an add-on therapy in children and adults (1). In addition, lamotrigine is approved as a mood stabilizer in affective disorders and is also frequently administered as augmentation antidepressant therapy (2–4). Similar to other anticonvulsants, lamotrigine is known to be able to provoke hypersensitive adverse reactions—even though less frequent than in older anticonvulsant medication such as phenytoin, carbamazepine, or phenobarbital (5–7). Considering its balance of efficacy and tolerability, lamotrigine ranks among first-line drugs for the treatment of bipolar disorders with the exception of acute manic episodes or conditions requiring rapid symptom control (3). Lamotrigine is eliminated via a hepatic route, whereby the metabolic inactivation through N-glucuronidation is primarily catalyzed by UGT1A4 (8). Interestingly, many polymorphisms could be identified in the coding regions of this enzyme, which result in corresponding changes in its catalytic activities. Moreover, a higher frequency of *UGT1A4* heterozygous mutations was reported in African populations compared with those in Caucasians (9). Indeed, the homozygous genotype was associated with better efficacy of lamotrigine (10).

## 2. Case description

In April 2022, a 32-year-old male inpatient of African ethnicity with a history of post-traumatic stress disorder (PTSD), recurrent major depressive disorder (MDD), and focal epilepsy was admitted to a psychiatric ward of a university department due to a current severe major depressive episode (MDE) that was accompanied by psychosomatic symptoms with predominant chronic pain. The patient presented physically fit apart from well-controlled arterial hypertension and the pronounced pain syndrome mentioned before. The psychosomatic character of the pain as primary etiology was assumed after the exclusion of physical causes and detailed anamnesis, which revealed a close temporal correlation between the reaggravation of pain and a recent stressful life event. Moreover, he received repeated neurosurgical interventions for traumatic brain injury between 2013 and 2020. At the time of admission, he received mirtazapine 60 mg, quetiapine 100 mg, pregabalin 600 mg, levetiracetam 1,000 mg, and 7 mg diazepam per day in addition to analgesics (dexibuprofen 800 mg and tramadol 400 mg) as well as lisinopril 20 mg. (CAVE: An off-label dose of 60 mg mirtazapine can only be administered after close therapeutic drug monitoring, because of CYP enzyme alterations necessitating higher doses and in special institutions enabling such therapeutic decisions. This was processed accordingly in an earlier admission of this patient.) Considering the current severe MDE and the known epileptic syndrome, lamotrigine at a dose of 25 mg once a day was added as an antidepressant augmentation strategy.

Two days after admission, respiratory deterioration of the patient was evident, whereby he became acutely unwell with shortness of breath, tachycardia (pulse rate 120/min in comparison with 105/min on admission), and oxygen saturation dropping to 55% on air during sleep. Subsequently, supplemental oxygen via a nasal cannula (starting at 2–3l/min) and inhalation therapy with short-acting beta-agonists were employed, and a comprehensive diagnostic workup was promptly initiated. On auscultation, the chest of the patient was clear. He had no rash, and his cardiovascular, abdominal, and neurological examinations remained without findings. His ECG was normal with no evidence of ischemia, and his blood pressure remained stable. His chest radiograph showed bipulmonal spotty opacities but no pleural effusion. Although the increased CRP (up to 8.73 mg/dl) was indicative of an infection, procalcitonin, markers of autoimmune response or rheumatologic origin, microbiology (blood cultures), and virology (nasal swab—including SARS-CoV-2, RSV, and influenza) results were negative.

## 3. Diagnostic assessment

**Figure 1:**
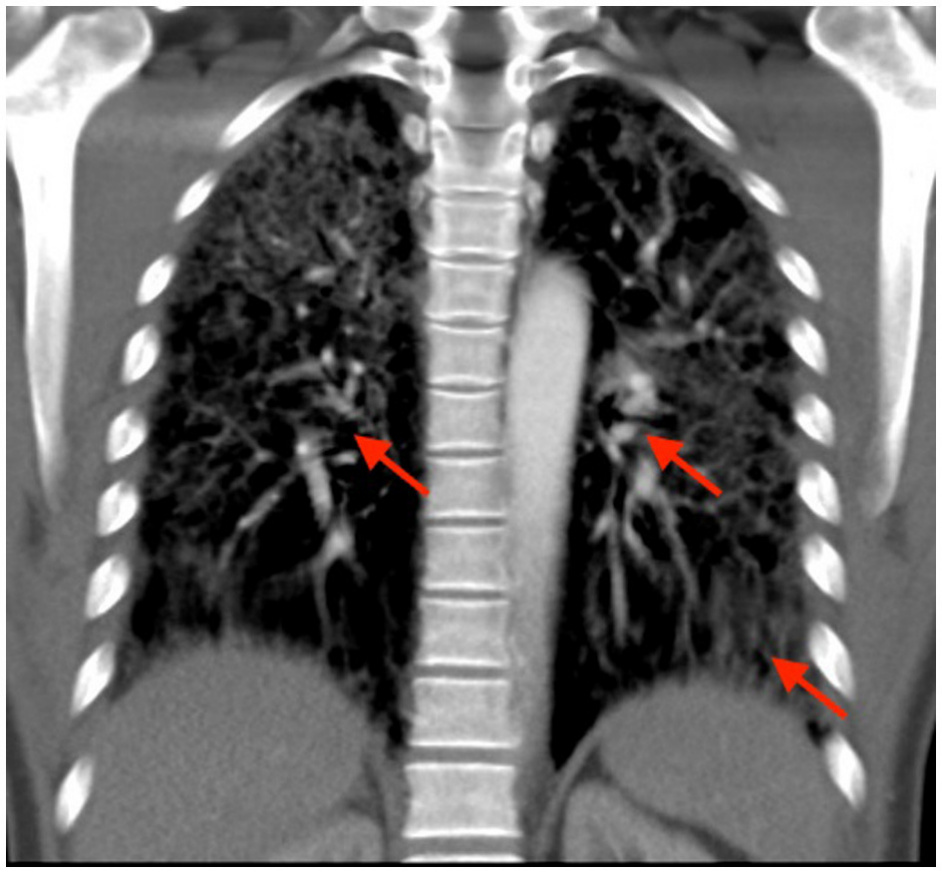
Computed tomography scan showing extensive bronchial wall thickening, interlobular septal thickening, and ground glass haziness on both lower lobes with bilateral hilar and mediastinal lymphadenopathy (pathological alterations marked with red arrows).

Upon suspicion of pneumonia, empirical antibiotic treatment with cefotaxime 2 g three times a day was administered. As the patient exhibited a constantly decreasing and insufficient oxygen saturation and required supplemental oxygen up to 9l/min (BGA: pO2 56 mmHg, pH 7.45), he was transferred to the intensive care unit (ICU) on the 6th day after admission, where high-flow oxygen nasal cannula therapy (50l/min, FiO2 70%) was provided and his antibiotic treatment was adapted (piperacillin 4 g/tazobactam 0.5 g three times a day and azithromycin 500 mg once a day) (until the arrival of the negative microbiology results) while psychopharmacotherapy was maintained unchanged. Additionally, a corticoid therapy (starting with 500 mg of prednisolone) was introduced once interstitial pneumonitis was suspected. The latter diagnosis was substantiated using chest computed tomography revealing extensive bronchial wall thickening, interlobular septal thickening, and ground glass haziness on both lower lobes with bilateral hilar and mediastinal lymphadenopathy (Figure 1).

**Figure 2:**
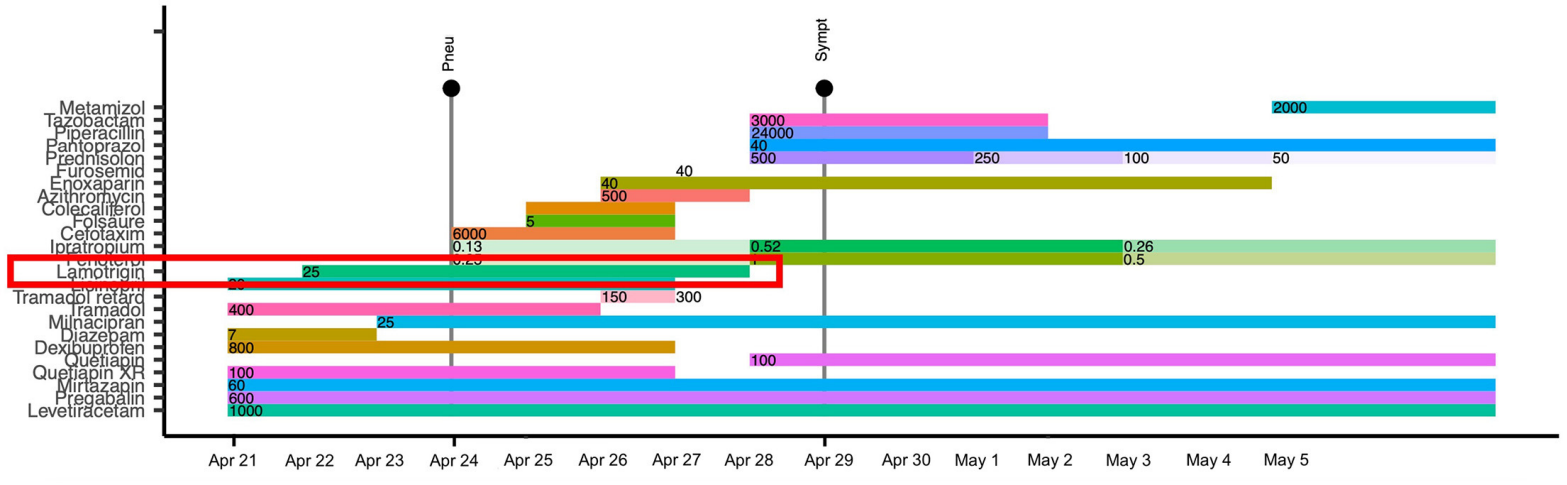
Graph showcasing a timeline of the case with the onset of pneumonitis and subsequent symptom reduction. Pneu, Onset of pneumonitis; Sympt, Improvement of symptoms; Apr, April; Folsäure, folic acid.

Because of an unremarkable history of substance abuse, familial predispositions, use of tobacco, or relevant exposures, the diagnostic workup described earlier, and as the initiation of lamotrigine augmentation was the only change in his medication at the onset of the respiratory symptoms, lamotrigine-induced pneumonitis was presumed as the most likely diagnosis. Accordingly, lamotrigine was discontinued (Figure 2), and the patient's oxygen demand decreased continuously. As the patient's general condition improved rapidly within a week and the patient was cardiorespiratory stable without supplemental oxygen, we were able to continue his treatment at the psychiatric ward. Subsequently, his corticoid therapy could be gradually reduced and discontinued. Concurrently, an antidepressant augmentation with quetiapine extended release 100 mg (two tablets 50 mg XR) and an antidepressant combination with milnacipran 200 mg per day were established. While a significant improvement of the depressive symptoms was achieved under the abovementioned treatment optimization, a clinically meaningful reduction in his psychosomatic symptoms manifesting as chronic pain was attained after pregabalin was increased to 1,200 mg per day. This is clearly an off-label dose that can only be titrated up according to kidney function (as pregabalin is eliminated predominantly renally), under close observation for potential side effects as well as therapeutic drug monitoring, and was tolerated well in the present case. Additionally, a reduction of perseveration in pain-related thoughts was successful after adding risperidone 2 mg at night. After treatment optimization, a final therapeutic drug monitoring revealed drug levels within the therapeutic range. Contemporaneously with the drug adaptations, the patient experienced relief from the burden of stressful life circumstances resulting in a significant reduction of not only depressive symptoms but also pain. Therefore, from a diagnostic point of view, the initially suspected diagnosis of a persistent pain disorder in the context of the psychiatric multimorbidity seemed confirmed.

## 4. Discussion

The present case report portrays severe respiratory deterioration that occurred in an adult African male inpatient immediately after the introduction of lamotrigine and that required intensive internal treatment. The consequently presumed diagnosis of a lamotrigine-induced interstitial pneumonitis is supported by previous singular observations on anticonvulsant hypersensitivity syndrome (ACHS), lamotrigine-associated pneumonitis, and the so-called drug reaction with eosinophilia and systemic symptoms (DRESS) syndrome with lung involvement (11–15). Previous reports portray both subacute and acute progressions of lamotrigine-induced pulmonary changes. Some describe concomitant skin alterations. In the present case, we were able to observe an acute progression over a couple of days and an isolated pulmonary affection. To exclude the possibility of a lamotrigine overdosage, even though lamotrigine was carefully introduced with a minimum daily dosage of 25 mg, we carried out genetic screening for variations in metabolizing enzymes and checked for possible drug interactions. Unfortunately, plasma concentrations were not measured during the acute phase. In addition to *UGT1A4*, we evaluated polymorphisms in *ABCG2, HLA-B*, and *SLC22A1*. For *HLA-B* (16) and *SLC22A1*, we found no relevant alleles associated with lamotrigine levels or toxicity according to pharmgkb.org. For the polymorphism *ABCG2 421C* > *A*, as found in our patient, an interaction with valproate on the steady-state disposition of lamotrigine with greater troughs was shown. However, the effects were contrary in patients receiving lamotrigine monotherapy with mildly lower troughs (17). Apart from a serious interaction of quetiapine and mirtazapine because of their effect on the QT interval, the interaction check did not show up any drug interactions for lamotrigine. As the diagnostic workup revealed unremarkable results, a hypersensitivity reaction to certain metabolites remained most likely as described in other hypersensitivity reactions to anticonvulsants (18). According to the Naranjo Scale, an algorithm for estimation of the likelihood of an adverse clinical event being actually caused by a specific drug, the pulmonary condition classifies as a probable adverse drug reaction (19). Moreover, the case was systematically documented and extensively discussed in the course of our national (ÖAMSP) and international (AMSP; Institut für Arzneimittelsicherheit in der Psychiatrie - Institute for Drug Safety in Psychiatry) psychopharmacotherapeutic conferences, whereby all medication-related adverse events are considered due to established protocols.

Our experience with a potentially life-threatening adverse drug reaction following the application of the largely well-tolerated lamotrigine might raise awareness for potential side effects under second-generation anticonvulsants including those that are deemed to be very rare (20). Although an altered lamotrigine metabolization seemed unlikely as a triggering factor in the present case and there are no data supporting a higher risk of lamotrigine intolerance in African populations, healthcare professionals might be encouraged to consider genetic testing, particularly on the occurrence of side effects and in the case of treatment resistance. Further research including the transparent documentation of side effects is needed to be able to estimate the risk of hypersensitivity reactions to second-generation anticonvulsants.

## Data availability statement

The original contributions presented in the study are included in the article/supplementary material, further inquiries can be directed to the corresponding author.

## Ethics statement

Ethical review and approval was not required for the study on human participants in accordance with the local legislation and institutional requirements. The patients/participants provided their written informed consent to participate in this study. Written informed consent was obtained from the individual(s) for the publication of any potentially identifiable images or data included in this article.

## Author contributions

VW wrote the case report including the first draft of the manuscript that was further elaborated and critically revised by LB. All authors were meaningfully involved in the performance of the reported therapy and the treatment of the patient, managed the literature search, and reviewed and approved the final manuscript.
